# Behavioral and Cognitive Impacts of Mindfulness-Based Interventions on Adults with Attention-Deficit Hyperactivity Disorder: A Systematic Review

**DOI:** 10.1155/2019/5682050

**Published:** 2019-04-04

**Authors:** Hélène Poissant, Adrianna Mendrek, Nadine Talbot, Bassam Khoury, Jennifer Nolan

**Affiliations:** ^1^Universite du Quebec à Montréal, Education and Pedagogy Department, Montréal, Quebec, Canada H3C 3P8; ^2^Bishop's University, Psychology Department, Sherbrooke, Quebec, Canada J1M 1Z7; ^3^Universite du Quebec à Trois-Rivières, Sciences Education Department, Trois-Rivières, Canada G9A 5H7; ^4^McGill University, Educational and Counselling Psychology, Montréal, Quebec, Canada H3A 1Y2

## Abstract

Mindfulness-based interventions (MBIs) are becoming increasingly popular as treatments for physical and psychological problems. Recently, several studies have suggested that MBIs may also be effective in reducing symptoms of attention-deficit hyperactivity disorder (ADHD). Most studies have examined the effectiveness in children, but there are now a sufficient number of individual treatment trials to consider a systematic review in adults. Majority of existing systematic reviews and meta-analyses only consider ADHD symptoms as an outcome, and most of them do not fully report potential biases of included studies, thus limiting considerably their conclusions. This is an important facet because some studies could be found ineligible to be included in future analysis due to their low quality. In this systematic review, we followed the PRISMA/PICO criteria and we thoroughly assessed the risks of bias for each of the selected studies according to Cochrane guidelines. We searched the available literature concerning MBIs in adult participants with ADHD using PsycINFO, PubMed, Scopus, and ERIC databases. In total, 13 studies conducted with 753 adults (mean age of 35.1 years) were identified as eligible. Potential moderators such as participants' age, ADHD subtypes, medication status, comorbidity, intervention length, mindfulness techniques, homework amount, and training of therapists were carefully described. Aside from measuring the symptoms of ADHD, outcome measures were categorized into executive/cognitive functioning, emotional disturbances, quality of life, mindfulness, and grade point average at school. According to presented descriptive results, all the studies (100%) showed improvement of ADHD symptoms. In addition, mindfulness meditation training improves some aspects of executive function and emotion dysregulation. Although these are promising findings to support treatment efficacy of MBIs for ADHD, various biases such as absence of randomization and lack of a control group may affect the actual clinical value and implications of the studies. Moreover, the relatively low quality of selection and performance criteria in several studies, as well as relatively high attrition bias across studies, call for caution before considering conducting further analysis.

## 1. Introduction

Mindfulness-based interventions (MBIs) have gained in popularity over the past decade. Clinical trials provide evidence of their effectiveness in the treatment of depression, anxiety, addictions, and other mental health problems. Most MBIs involve three somatically focused meditative techniques (body scan, sitting meditation, and mindful yoga) that are thought to help participants cultivate nonjudgmental, mindful awareness of present-moment experience. Recently, several studies have suggested that MBIs may also be effective in reducing symptoms of attention-deficit hyperactivity disorder (ADHD). Most studies have examined the effectiveness in children. There are now a sufficient number of individual treatment trials to consider a systematic review in adults. To our knowledge, only Cairncross and Miller [1] have conducted a meta-analysis (six studies in children and four in adults) on ADHD to measure the impact of MBIs on symptoms of ADHD. The authors only considered publication bias in their study. The present systematic review goes further by exploring in detail seven types of biases according to Cochrane guidelines. This is an important aspect of the present review because it allows identifying studies with poor quality.

This systematic review examines if MBIs are effective treatments of attention-deficit hyperactivity disorder (ADHD) in adults. ADHD is characterized by marked behavioral symptoms such as inattention and/or hyperactivity and acting impulsively. The prevalence of the disorder is about 3-4% in adults, and it is higher in males than in females. ADHD often exists simultaneously with other conditions, such as anxiety, depression, and personality disorders. The most common treatment of ADHD consists of administration of psychostimulant medications. However, the pharmacotherapy is not always effective and is associated with various side effects. Thus, MBIs represent a much-welcomed addition to available treatments or a stand-alone therapy.

Comprehension of the mechanisms mediating the effectiveness of MBIs in ADHD at both the behavioral and neuronal levels has greatly improved. Thus, three large neural networks have been implicated both in ADHD and in mindfulness meditation: the default mode network, salience network, and central executive network [2–7]. Because of space constraints, we concentrate in the present review only on the behavioral level, considering the effects of MBIs on hyperactivity, emotion dysregulation, deficits in attention and executive function (EF), and other problems. Most of these dysfunctional behaviors seem to improve following MBIs. The improvement of these behaviors in turn contributes to the general well-being of adults with ADHD.

However, due to the heterogeneous presentation of ADHD, it is important to document the interindividual differences of individuals with ADHD. These differences between individuals with ADHD may affect the success of MBIs in reducing symptoms. For example, there is more evidence to suggest that MBIs are effective in reducing inattention, so perhaps the intervention is more helpful for individuals with ADHD with a predominantly inattentive type [1]. Adults being more often characterized as inattentive (instead of hyperactive) may react in a different manner to MBIs compared to children. Other variables could also affect whether MBTs are effective in improving functioning in individuals with ADHD. For example, it is unclear how factors such as the length of intervention, mindfulness techniques, amount of homework, homework compliance, and training of the therapist affect the outcome of therapy [1]. These are important elements that should be taken into account. Moreover, besides symptoms of inattention and hyperactivity, other indicators of efficacy of MBTs had been reported, namely, EFs, emotional disturbance, quality of life, and academic performance. We consider these additional elements useful in supporting the portrayal of ADHD.

The present systematic review attempts to provide more evidence for the use of MBIs in adults with ADHD by means of investigation into several variables and characteristics that may moderate the effectiveness of MBIs. It is important for us to report these elements so that the relationship between these characteristics and intervention effectiveness can be better understood. Moreover, incorporation of an exhaustive analysis of biases with an assessment of the quality of studies is presented here as an essential element of the systematic review.

## 2. Methods

### 2.1. Eligibility Criteria

In order to conduct the systematic review, we used the criteria from PRISMA-P [8]. These criteria allow following a stepwise methodology in conducting and reporting the outcomes of the systematic review. A data sheet based on the PRISMA-P protocol was designed and comprised information extracted from each selected study based on (1) research design (including RCT, N-RCT, the presence of a waiting list, pre-posttest, follow-up, and baseline), (2) characteristics of participants (including number, age, gender, diagnosis with subtype, comorbidity, and medication), (3) characteristics of the intervention (including type, description, length, identity of therapists, and their experience), and (4) characteristics of outcomes (ADHD symptoms, executive functioning, emotional disturbance, mindfulness, and quality of life).

We limited inclusion to peer-reviewed empirical published studies that examined effects of meditation or mindfulness-based interventions (MBIs) on symptoms of ADHD. All studies were published in English. We excluded books, reviews, meta-analyses, qualitative, psychometric, or single-case studies, and duplicates.

### 2.2. Information Sources and Search

We consulted PsycINFO, PubMed, Scopus, and ERIC databases from the first available date until June 2018 (+reference lists of previous reviews). By the end of the search, the selected studies cover a period from 2002 to 2018. The search terms “ADHD AND meditation OR mindfulness” and associative terms (e.g., impulsivity, inattention, and hyperactivity) were considered for inclusion. Impact of MBIs was extended to cognition, EF, and brain structure alterations. We included randomized and nonrandomized control trials (RCT and N-RCT, respectively), pre-posttest (within-group) studies, clinical trials, prospective or follow-up studies, and single- or double-blind studies. Studies were excluded if they (1) were conducted with children, (2) did not include a mindfulness- or meditation-based treatment, (3) did not include a group of ADHD or ADD or hyperactivity disorders, (4) did not examine treatment effects, (5) did not report clinical outcomes, and (6) described solely mindfulness or meditation instructions. We included different forms of MBIs as long as the intervention contained significant elements of mindfulness. We also excluded “gray literature,” reports, and unpublished studies. By the end of the selection process, we included thirteen studies conducted with adults, young adults, and college students. The full electronic process search strategy for our databases is described below.

### 2.3. Risk of Bias in Individual Studies

We looked for risk of bias in each individual study. Thus, we designed and adapted a classification to report potential bias for each individual study using the Cochrane Collaboration [9] recommendations. Biases included (1) “sequence generation” (e.g., Is the allocation sequence acceptably generated? “YES” if explicitly mentioned that the “patients were randomly allocated”), (2) “allocation concealment” (e.g., Is the allocation acceptably concealed? “YES” if participants and researcher could not foresee assignment because of an explicit mention of a method to conceal allocation), (3) “blinding of participants, personnel, and outcome assessors” (e.g., Is knowledge of the allocated treatment plenty prevented during the study? “YES” in case of blinding or if the authors judged improbably that the outcome measurement was influenced by no blinding, and (4) “selective outcome reporting” (e.g., Are partial outcome data adequately addressed? “YES” if explicitly mentioned that nonmissing outcome data or reasons for missing outcome data had little impact on outcomes). The authors' judgments involved answering specific questions for each query and providing a detailed entry addressing the sources of bias. In all cases, an answer “YES” indicates a low risk of bias, an answer “NO” indicates a high risk of bias, and an answer “unclear” indicates an uncertain risk of bias (p. 196, [9]).

*Selection bias* comprises random “sequence generation” (1) and “allocation concealment” (2) (p. 196, [9]). According to the criteria proposed by the Cochrane Collaboration [10], a study was rated low risk on random sequence generation if the method used to allocate sequence produces equivalent groups. If random sequence generation was not described in sufficient detail but the study was described as randomized and the groups were equivalent, we rated the risk unclear. The studies, which have not fulfilled either condition, were rated high risk. Regarding allocation concealment, we rated a study low risk when its method to conceal the allocation sequence could not be predicted in advance of or during intervention. If allocation concealment was not described with sufficient accuracy to allow an appreciation of whether it could be foreseen but participants would not necessarily identify the group to which they belong (i.e., treatment or control), the risk was rated unclear. The remaining studies, which have not fulfilled either condition, were rated high risk.

*Performance bias and detection bias* (3) reflect the blinding of participants and personnel (e.g., facilitators or trainers) and reflect the blinding of assessors to the condition. We considered studies low risk if they described the blinding of outcome assessors and used only self-report subjective measures and/or objective measure (e.g., neuropsychological measures). Unclear risk corresponded to self-report measures but nonblinding of assessors. For the nature of interventions in this study (MBIs), it is not habitual, nor always advantageous, to blind personnel or participants. Therefore, we expected studies to have an elevated risk for performance and/or detection bias.

*Attrition bias* (4) is based on the recommendation by Higgins et al. [10] to rate studies with above 20% attrition of participants as high risk. An attrition rate lower than 20% where the groups (i.e., treatment and control) are equivalent yields a low risk. Thus, studies were rated high risk if the attrition rate was greater than 20% and the authors did not use any analyses to compensate for the missing data. Studies were rated unclear risk if the authors did not explicitly provide attrition rates or the computation of the attrition was not possible using the provided data.

*Reporting bias* refers to reporting the outcome data partially or omitting to report scales (or subscales) that may lead to a bias. We decided to rate the study low risk if all the scales and subscales were reported. We rated the study unclear risk when subscales were not fully reported or it was unclear whether omitting to report the subscales led to a bias.

*Other biases* included a researcher's allegiance and funding source. These biases addressed the authors' role in the study development or implementation, as well as acknowledgement of any conflict of interest. Studies were rated high risk when authors were actively involved in delivering interventions, evaluating participants, or conducting any other aspects of the study. Studies were rated low risk when authors were not involved in conducting the study. Studies were rated unclear when the authors did not report their involvement and information from the paper did not suggest their involvement. Regarding funding, studies were rated high risk when sources of funding can cause a conflict of interest, low when the study was not funded or when the sources of funding were disclosed with a nonconflict of interest statement, and unclear when the sources of funding were not reported.

Discussions about the judgment ratings were provided in an iterative way until consensus about the ratings was reached between judges (H.P., B.K., and A.M.). Prior to these discussions, the rating coauthors familiarized themselves with a series of articles and a document containing specific instructions and examples of rating the studies from the Cochrane Collaboration's tools.

### 2.4. Summary Measures and Additional Analyses

The results of the present systematic review are first presented in a narrative manner. Tables 1 and 2 give an overview of a PICO description of each individual study considering two separate types of research design: within and between subjects (see Tables 1 and 2, respectively). For the bias analysis, appreciation of the quality of each study was converted into numeric variables with quality scores ranging from 0 (high risk) and 1 (unclear risk) to 2 (low risk) on each of the seven bias evaluation measures (the maximum score of quality for each study is 14).

## 3. Results

### 3.1. Study Selection

**Figure 1:**
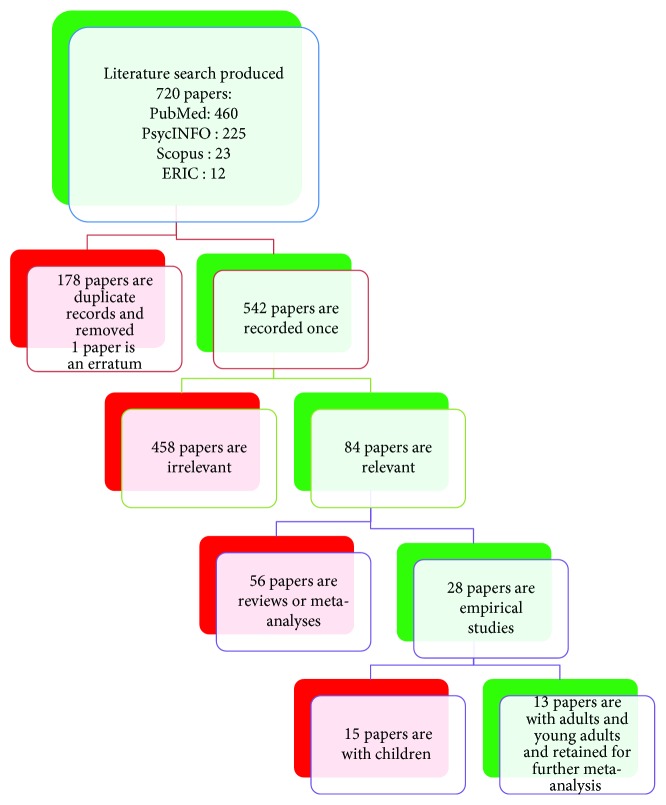
Flow chart of the eligibility criteria.

The final literature search resulted in 720 studies: PsycINFO (*n* = 225), PubMed (*n* = 460), Scopus (*n* = 23), and ERIC (*n* = 12). An Endnote file was first created, and abstracts of all articles were saved on an electronic file for further examination. The search was conducted in two consecutive sessions from October 2016 to January 2017 and later updated from April 2018 to July 2018 resulting in the incorporation of two new articles (with the help of N.T.). The first step consisted of the elimination of 178 duplicates (+1 erratum). The final study selection was based on eligibility assessment from two independent reviewers (H.P. and A.M.). Disagreements were resolved through discussions. After reviewing the abstracts of the 542 remaining studies, 458 studies were classified as irrelevant (erratum, theoretical paper, qualitative study, no ADHD group, program development, case study, study protocol, no treatment, and no quantitative outcome). From the remaining 84 studies, 56 were reviews or meta-analyses; therefore, they were eliminated (but we examined their reference lists). Among the 28 remaining empirical studies, 15 were conducted with children, adolescents, and/or their parents; therefore, they were excluded. Thirteen studies conducted with adults, young adults, and college students corresponded to all the selection criteria; therefore, they were included. A detailed illustration of the study selection process is found in Figure 1. The systematic review of the thirteen studies selected is presented qualitatively (narrative review).

### 3.2. Study Characteristics and Results of Individual Studies

Each of the following study characteristics (e.g., study size, PICOS, and follow-up period) and results of individual study are presented in Tables 1 and 2. Outcomes in terms of benefits are presented in a narrative manner for each study in the form of a simple summary data for each intervention group. For briefness, we choose not to list the effect size (ES) for every outcome (e.g., all tests and subtests and T1-T2-T3-T4, totalizing 470 ESs for all articles). Rather, a summary of effect sizes (ESs) is given for each study with a main focus on symptom outcomes and posttest (T2) or follow-up. Most ESs were retrieved directly from the articles. However, for Zylowska et al. [11], Schoenberg et al. [12], and Bachmann et al. [13], the ESs were not provided by the authors. Therefore, for consistency, we calculated the ESs using Comprehensive Meta-Analysis (CMA) software. Since Bachmann et al. [13] did not find evidence for a significant main effect of type of treatment (MAP vs. the psychoeducation comparison group), we did not calculate any ES for this study (see below).

In an early pilot study, Hesslinger et al. [14] evaluated a training based on dialectical behavior therapy (DBT) to suit the special needs of adult patients with ADHD. The overall treatment goal was that patients would “control ADHD rather than being controlled by ADHD.” Prior to and following group therapy, symptoms were assessed using self-rating scales of ADHD-CL (from DSM-IV), a short version of the SCL-16 to assess nervousness, memory deficits, carelessness, excitability, emotional outburst, self-reproach, difficulties to start, inferiority complex, sleep disturbances, concentration deficits, feeling of tension, embarrassment, exertion, restlessness, worthlessness, thinking something is wrong with comprehension, and the BDI [15]) to assess depressive symptoms (see the appendix for the complete names of tests). In addition, neuropsychological testing was performed at baseline and following treatment, including a verbal and letter fluency test, the Stroop test indicating mental speed and inhibitory EFs, the digit symbol subtest to evaluate divided attention, a test of continuous attention, the d2-Test measuring selective attention, and tests measuring mental control, digit span, and visual memory span indicating short-term memory, working memory (WM), and general attentional capacities [16]. The DBT treatment resulted in mild to moderate improvements on all the measured symptoms and even greater improvements in neurocognitive function (ESs ranging from 0.99 to 2.22).

Subsequently, Zylowska et al. [11] enrolled adults and adolescents with ADHD in the mindful awareness program (MAP) [17, 18] adapted to meet the challenges of ADHD symptoms, including a psychoeducational component. Self-report scales of ADHD, depression, and anxiety symptoms and several cognitive tests were administered to participants during pre- and postintervention sessions. ADHD symptoms were assessed via the ADHD Rating Scale IV [19] that measures the severity of symptoms. Self-reports of anxiety and depression were assessed using the BAI [20] and BDI. Attention was assessed using the ANT [21] measuring three aspects of attention: alerting (maintaining a vigilant state of preparedness), orienting (selecting a stimulus among multiple inputs), and conflict (prioritizing among competing tasks). Authors also used the Stroop test and measure of attentional conflict with the ANT [22]; the Trail Making Test [23], which assesses set-shifting and inhibition; the Digit Span Test, which measures WM; and the vocabulary subtest (WAIS-R) [24]. Improvements were found in depression and anxiety as well as improvements in ADHD self-reported symptoms (ESs ranging from 0.50 to 0.93) and measures of attentional conflict and set-shifting after the training (ES = 0.93 and 0.43, respectively).

Later on, Mitchell et al. [25] tested the impact of MAP for adults with ADHD on symptoms, EF, and emotion dysregulation. Adults were stratified by ADHD medication status and then randomized into a group-based mindfulness treatment or waitlist group. The authors observed large effect sizes in improvement of self-reported and clinician ratings of ADHD symptoms (ESs ranging from 1.35 to 3.14) and EF (ESs ranging from 1.45 to 2.67) as well as self-reported emotion regulation (ESs ranging from 1.27 to 1.63), for the treatment group relative to the waitlist group. EF self-report scales included the DEFS [26] and the BRIEF-A [27], which consists of nine scales: Inhibit, Shift, Emotional Control, Self-Monitor, Initiate, WM, Plan/Organize, Task Monitor, and Organization of Materials. Emotion dysregulation was assessed by the DERS [28] and the DTS [29]. The DERS assesses how often emotionally dysregulated behavior occurs. Additional EF tasks were also administered: the ANT, the CPT [30] to measure response inhibition, the Digit Span Test [31] to measure WM, and the Trail Making Test to assess attentional set-shifting and inhibition.

Edel et al. [32] recruited adults with ADHD and nonrandomly assigned them to mindfulness-based training (MBT, including elements of DBT) or a skills training group (ST). The WRI [33] and scales covering the inattention and hyperactivity/impulsivity symptoms (DSM-IV, [34]) were used for pre- and postassessment. The WRI is an expert-rated scale comprising symptom domains such as attentional difficulties, hyperactivity/restlessness, (hot) temper, affective lability, emotional overreactivity, disorganization, and impulsivity. General linear models with repeated measures revealed that both programs resulted in a similar reduction of ADHD symptoms. The effect sizes were in the small-to-medium range (ESs ranging from 0.06 to 0.49). However, some degree of decrease in ADHD symptoms (30%) was more prominent for the MBT participants since 30.8% of them showed improvement compared to 11.5% of the ST participants.

Fleming et al. [35] conducted an RCT evaluating DBT training adapted for college students with ADHD randomized to receive either DBT or skills handouts. ADHD symptoms, EF, and related outcomes were assessed at baseline, posttreatment, and 3-month follow-up. Authors used the BAARS-IV (based on DSM-5 criteria) to assess ADHD symptoms. Self-report of current symptoms was used as a primary outcome measure. EF was assessed with the BADDS, a self-report questionnaire [36] that yields scores on categories of EF: organization and prioritization, focused and sustained attention, regulation of alertness and sustained effort, affect modulation, and WM. Anxiety and depressive symptoms were assessed with self-report measures of BAI and BDI-II [36]. The CPT-2 [37] provides assessment of sustained attention, inhibition, and response variability [38, 39]. Overall, participants receiving DBT group skills training showed greater treatment response rates (59-65% vs. 19-25%) and higher clinical recovery rates (53-59% vs. 6-13%) on ADHD symptoms (ES = 0.84 at follow-up) and EF (ES = 0.81 at follow-up).

In a similar approach, Cole et al. [40] addressed training skills by means of cognitive behavioral therapy (CBT) or DBT. They assessed the benefits of the program to reduce residual symptoms. Patients with ADHD who were poor responders to medication were enrolled in a one-year program where they received individual therapy, associated with group therapy with different modules that included mindfulness (along with emotion regulation, interpersonal effectiveness and distress tolerance, impulsivity/hyperactivity, and attention). Each subject was assessed at baseline, at 3 and 6 months, and at the end of the treatment for ADHD severity with the ASRS v1.1, for depression with BDI-II, for hopelessness with the BHS, for anger experience, expression, and control with STAXI [41], and for impulsivity with BIS-11. The ADHD patients were compared with ADHD patients on a waiting list. Overall, the treatment was associated with significant improvements in almost all dimensions. The most significant changes were observed with large to moderate effect sizes for depression (ES = −0.84) followed by ADHD severity (ES = −0.63) and hopelessness (ES = −0.52).

Morgensterns et al. [42] also used DBT for adults with ADHD in an outpatient psychiatric context. The treatment uses elements such as acceptance, mindfulness, functional behavioral analysis, and psychoeducation to target problems common in ADHD. Self-rating scales were administered at baseline before the first session (T1), posttreatment (T2), and 3-month follow-up (T3). Self-rating of current ADHD symptoms was measured by the Current ADHD Symptom Scale-Self-Report Form [43] that contains three parts: (1) the symptoms for ADHD, (2) impairment in major life areas, and (3) symptoms of irritability and aggressiveness. Moreover, participants completed self-rating questionnaires for assessing symptoms of psychiatric comorbidity: the BDI and the BAI. The main results indicated that approximately 80% of the participants attended at least two-thirds of the sessions. ADHD symptoms (ES = 0.22) and functional impairment (ES = 0.15) in everyday life were reduced. The results were stable at 3-month follow-up. Variables such as age, comorbidity, ADHD medication status, and IQ level did not predict outcomes.

A study from Bueno et al. [44] addresses the impact of MAP on affective problems and impaired attention. Adults with ADHD and healthy controls underwent MAP sessions while similar patients and controls did not undergo the intervention. The authors evaluated MAP-induced changes in mood and attention using several measures: (1) the ASRS for symptom assessment, (2) the BDI for attitudes related to depression, (3) the STAI to describe how people feel at a particular moment, and (4) the PANAS-X to assess feelings or moods. Combinations of these ratings yield to “higher-order affective levels” (positive affect and negative affect) and “lower-order affective levels” (fear, sadness, guilt, etc.). Attention was evaluated using the ANT and the CPT-2, before and after intervention. The authors found that MAP enhanced sustained attention (ANT) and detectability on the CPT-2 and improved the mood of patients and healthy controls with overall medium effect sizes (*g* > 0.5) to large effect sizes (*g* > 0.8). Because of mixed results regarding the enhancement of attentional performance (not all attentional measures were found significant), the authors call for more studies that address the efficacy of mindfulness meditation for ADHD in terms of its impact on EF.

In a recent study, Bachmann et al. [13] evaluated the impact of MAP on neurocognitive performance in adults with ADHD. The authors performed a RCT to investigate WM with an *n*-back task during fMRI before and after an 8-week mindfulness intervention. ADHD symptoms were assessed using the self- and observer-rated Conners Adult ADHD Rating Scales (CAARS). The researchers found a significant decrease in ADHD symptoms and significant improvement in task performance in both the MAP and the psychoeducation comparison group post- versus preintervention but did not find evidence for a significant main effect of treatment or a significant interaction effect on any ADHD symptoms (self- and observer-rated) nor on task performance (WM). Results also revealed significant increased brain activation after MAP in the bilateral inferior parietal lobule, right posterior insula, and right precuneus. A decrease in self-rated “inattention/memory problems” after MAP compared to baseline was associated with stronger activation in parts of the left putamen, globus pallidus, and thalamus.

Hepark et al. [45] also looked at the efficacy of an adaptation of mindfulness-based cognitive therapy (MBCT) on core ADHD symptoms and EF. Adults with ADHD were randomly allocated to MBCT or waitlist. Outcome measures included investigator-rated ADHD symptoms, self-reported ADHD symptoms, EF, depressive and anxiety symptoms, and patient functioning. Symptoms (total ADHD, inattention, and hyperactivity/impulsivity scores) were assessed by a clinician with the CAARS-INV [46] as well as with the self-report of the CAARS-SR [46]. EF was assessed using the BRIEF. The BDI-II was used to assess the presence of depression symptoms. The Dutch version of the STAI [47] was administered. The findings indicate that MBCT resulted in a significant reduction of ADHD symptoms as assessed by the investigator or self-reported (Cohen's *d* = 0.78 and 0.64, respectively). Significant improvements in EF were also found (Cohen's *d* = 0.93). However, no improvements were observed for depressive and anxiety symptoms.

Schoenberg et al. [12] looked at the effects of MBCT on neurophysiological correlates (event-related potentials (ERPs)) of performance monitoring in adults with ADHD. Half of patients were randomly allocated to MBCT, and the other half to a waitlist control. Inattention and hyperactivity-impulsivity ADHD symptoms, psychological distress, and social functioning were assessed. Clinical scales (the CAARS-S:SV) were administered pre- and post-MCBT (or waiting list (WL)). Participants also completed a standard visual continuous performance task (CPT-X). Examining results for CAARS-S:SV indicated reduced inattention, hyperactivity/impulsivity, and global ADHD index symptoms pre to post-MBCT (ESs ranged between 0.49 and 0.93). As expected, the main effect of treatment was evident for CPT-X repeated-measures ANCOVAs comparing accuracy score data indicated that the number of false alarms (FA) significantly decreased pre to post in the MBCT group alongside a significant slowing in reaction times.

Gu et al. [48] conducted a clinical trial to assess MBCT efficacy in the treatment of ADHD in college students. Undergraduates with ADHD between ages 19 and 24 were randomized either to receive MBCT or to be put on a waitlist. ADHD symptoms, neuropsychological performance, and related outcomes were assessed pre- (T1) and posttreatment (T2), as well as at the 3-month follow-up (T3). Clinical assessment was conducted with the CAARS-S:SV to assess the extent of ADHD symptoms. Anxiety and depressive symptoms were measured with the BAI and the BDI-II. In addition, academic performance was collected (participants' GPA) using an official transcript. The authors tested the participants' neuropsychological performance (MAAS) and attentional networks with the ANT [49]. At follow-up, results revealed that participants receiving MBCT showed greater treatment response rates (57%-71% vs. 23%-31%) and symptom reduction (ES = 1.26). Participants also experienced less anxiety and depression (ES = 0.75 and 0.53, respectively) than those on the waitlist. Moreover, MBCT participants showed greater improvement on most neuropsychological performance and attentional scores (ES for MAAS = 1.30, ES for ANT subscales ranging from 0.19 to 1.19).

In a recent study, [50] investigated the efficacy of MBCT+treatment as usual (TAU) versus TAU only in reducing core symptoms in adults with ADHD. Participants were randomly assigned to MBCT+TAU, an 8-weekly group therapy including meditation exercises, psychoeducation, and group discussions, or TAU only, including pharmacotherapy and/or psychoeducation. Outcomes were ADHD symptoms rated by blinded clinicians (CAARS-INV) and self-reported (CAARS-S), EF, mindfulness skills, self-compassion, positive mental health, and general functioning (see details in Table 2(c)). Outcomes were assessed at baseline, posttreatment (T1), and 3- and 6-month follow-up (T2 and T3, respectively). In MBCT+TAU patients, a significant reduction of clinician-rated ADHD symptoms (CAARS-INV) was found at posttreatment (T1) (ES = 0.41) and was maintained at the 6-month follow-up. MBCT+TAU patients compared with TAU patients also reported significant improvements in self-reported ADHD symptoms (ES = 0.37, 0.71, and 0.79 at T1, T2, and T3, respectively), mindfulness skills, self-compassion, and positive mental health up to the 6-month follow-up. Patients in MBCT+TAU reported improvement in executive functioning (EF) but only at the 6-month follow-up. A significant group x time interaction showed that EF further improved over time in MBCT+TAU compared with TAU resulting in an effect size of *d* = 0.49 at the 6-month follow-up. The authors concluded that MBCT might be a valuable treatment option alongside TAU for adult ADHD aimed at alleviating symptoms.

### 3.3. Synthesis of Studies

For convenience, we divided the above studies (*n* = 13) according to the two main research designs: within-group (Tables 1(a)–1(c)) and between-group (Tables 2(a)–2(c)). Three studies used a within-group design (with two or more time points) while the remaining studies used different between-group designs (N-RCT, RCT). All studies, except one [44], did not include a healthy control group. Follow-up evaluations varied from none to three or six months.

#### 3.3.1. Participants

The sum of participants with ADHD was 753 with a mean age of 35.1 years (18-65 y/o). About half of participants were males (47.7%). The combined and inattentive subtypes of ADHD were the most predominant. Most participants were on medication, with psychostimulants like methylphenidate (MPH) being the most frequently reported. Comorbidity was present in all studies, with major depressive disorders and mood disorders being frequently reported. A summary of participants' characteristics is presented in Tables 1(a) and 2(a).

#### 3.3.2. Intervention

Our definition of MBI intervention included mindfulness and/or meditation as a principal or a partial component of the intervention. That included various adaptations of (1) dialectical behavior therapy (DBT), (2) mindful awareness program (MAP), and (3) mindfulness-based/cognitive training (MBT/MBCT). The duration of treatment varied considerably across studies (from six to 96 hours, mode value of 20 hours). The presence or absence of homework also accounted for variability between studies. Therapists included clinical psychologists, psychology graduate students, mindfulness instructors, practitioners, group leaders, ADHD researchers, nurses, and psychiatrists. A summary of intervention characteristics is presented in Tables 1(b) and 2(b).

#### 3.3.3. Outcomes

Besides the measures of ADHD symptoms (inattention and hyperactivity), outcome measures can be categorized into executive/cognitive functioning, emotional disturbance, quality of life, mindfulness, and grade point average at school.

*(1) ADHD Symptoms*. Prior to and following treatment, researchers used different self-rating scales to assess symptoms of ADHD. Among the most frequently used self-report scales were the following:

1. The Conners' Adult ADHD Rating Scale (CAARS-SR/CAARS-S:SV) from Conners et al. [46, 51] ([12, 13, 45, 48, 50], *n* = 5)
2. The Adult ADHD Self-Report Scale (ASRS v1.1) from Kessler et al. (2005) ([40, 44], *n* = 2)
3. The Attention-Deficit Hyperactivity Disorder Checklist (ADHD-CL) from DSM-IV (1994) ([14, 32], *n* = 2)

The complete list of scales is available in Tables 1(c) and 2(c).

*(2) Executive/Cognitive Functioning*. As additional measures of outcomes, the most frequently used tests for executive/cognitive functioning were as follows:

- Objective tasks
  1. Various versions of Attention Network Test (ANT) [21, 22, 49] ([11, 25, 44, 48], *n* = 4)
  2. Various versions of the Conners' Continuous Performance Test (CPT/CPT-2/CPT-X) from Conners [30, 37] ([12, 25, 35, 44], *n* = 4)
  3. The TMT (*n* = 2)
  4. The Stroop test indicating mental speed and inhibitory EFs ([11, 14], *n* = 2)
  5. The digit span, vocabulary, memory scale, and digit symbol subtests from the Wechsler Adult Intelligence Scale-Revised (WAIS-R) [24, 31] ([11, 14, 25], *n* = 2)
  6. *n*-back ([13], *n* = 1)
- Subjective questionnaire
  1. The Behavior Rating Inventory of Executive Functioning-Adult Version (BRIEF-A) from Roth et al. [27] ([25, 45, 50], *n* = 3)

The complete list of outcome measures is available in Tables 1(c) and 2(c).

*(3) Emotion Disturbance*. Self-reports of anxiety, depression, and other emotional disturbances were often assessed using the following:

1. The Beck Depression Inventory (BDI/BDI-II/BDI-II-NL) from Beck et al. [15] ([11, 14, 35, 40, 42, 44, 45, 48], *n* = 8)
2. The Beck Anxiety Inventory (BAI) from Beck et al. [20] ([11, 35, 42, 48], *n* = 4)
3. The State-Trait Anxiety Inventory (STAI) from Van der Ploeg [47] ([44, 45], *n* = 2)
4. The Outcome Questionnaire (OQ 45.2) from Lambert et al. (1996) ([12, 45], *n* = 2)

The complete list of outcome measures is available in Tables 1(c) and 2(c).

*(4) Mindfulness*. Mindfulness was assessed using the following self-report questionnaires:

1. The Mindful Attention Awareness Scale (MAAS) from Brown and Ryan (2003) ([32, 42, 48], *n* = 3)
2. The Kentucky Inventory of Mindfulness (KIMS) from Baer et al. (2004) ([12, 40, 45], *n* = 3)

At two occasions, authors used the Five Facet Mindfulness Questionnaire (FFMQ) from Baer et al. (2006) ([35, 50], *n* = 2).

*(5) Quality of Life and Others*. Self-report questionnaires or semistructured interviews (QFS) were used in most studies to evaluate the level of functioning and quality of life:

1. The ADHD Quality of Life Questionnaire (AAQoL) from Brod et al. (2006) ([35, 42, 44], *n* = 3)

Other less frequently used measures, such as OQ 45.2 (*n* = 2), are listed in Tables 1(c) and 2(c).

### 3.4. Risk of Bias within and across Studies

**Figure 2:**
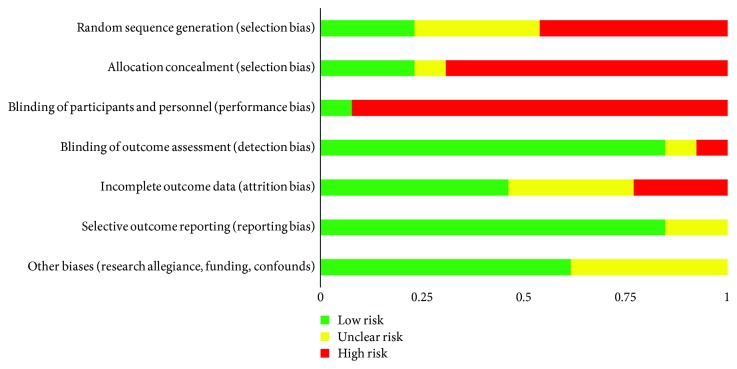
Methodological quality graph: a review of the authors' judgements about each methodological quality item presented as percentages across all included studies.

**Figure 3:**
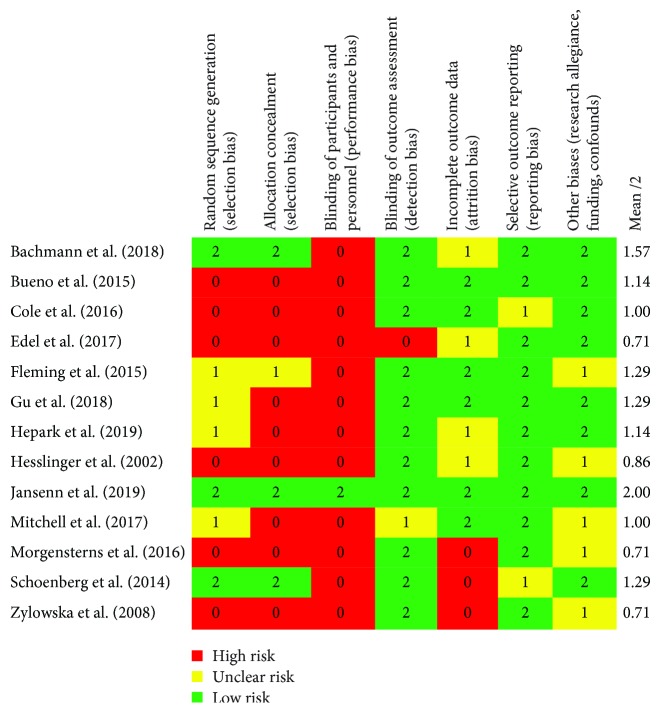
Methodological quality summary: a review of the authors' judgments about each methodological quality item for each included study.

After characterizing each study according to PICO, we then evaluated each study (*n* = 13) on the seven categories of bias established by the Cochrane Collaboration. As alluded earlier, this analysis of bias gives us a useful complement of information besides the sole calculation of effect sizes. Slight differences in interpretation of bias were discussed and solved between the judges (HP, BK, and AM). Assessment of risk of bias was rated high (red), uncertain (yellow), or low (green) for each individual study and category (see Figure 2), then compiled into percentages of studies that fall into high, uncertain, or low risk on each category of bias (see Figure 3).

### 3.5. Selection Bias

Only three studies were rated low risk on that criterion [12, 13, 50]. Four studies (31%) were rated unclear risk because of insufficient data on sequence generation. The remaining six (46%) studies were rated high risk. Regarding allocation concealment, the same three studies were judged as low risk because the occultation of the allocation sequence could not be predicted. Only one study was judged as unclear risk because participants would not necessarily identify the group to which they belong (i.e., treatment or control). The remaining nine (69%) studies did not fulfill either conditions and were considered high risk.

### 3.6. Performance and Detection Bias

Not surprisingly, the vast majority of studies (92%) in this review were rated high risk for performance bias (blinding of participants and of personnel). Only [50] satisfied this criterion according to our interrater judgment. Detection bias reflects the blinding of assessors to the treatment condition. Overall, eleven studies (85%) were judged as low risk of detection bias (blinding of assessors), one study as high risk, and one as unclear risk as it included self-report measures but nonblinded assessors.

### 3.7. Attrition Bias

According to the application of the 20% cut-off criteria, six studies (46%) in this review were considered low risk of attrition bias. Three studies (23%) were rated high risk (attrition rate > 20%, high differential attrition, and no imputation of missing data). The remaining three studies had no sufficient description of attrition (or impossibility to compute the attrition rate) and thus were judged as having unclear risk.

### 3.8. Reporting Bias

Most of the studies (11/13) were rated low risk since the outcome data were reported on all used scales and subscales. Two other studies were rated unclear risk as subscales were not fully reported.

### 3.9. Other Biases and Limitations

Overall, eight studies (62%) were rated low risk, while the five remaining studies were judged as unclear risk. More precisely, we could identify an author's role in the study development and/or implementation (e.g., delivery of the intervention) in only three (23%) out of the 13 studies. In four of the studies (31%), the authors identified a funding source. Other studies were not funded or did not report the funding source. Other limitations that were reported by the authors of each paper were included as an additional source of information (in narrative form) but were not rated (see supplementary materials (available here)).

After conversion of the high, uncertain, and low risk scores into numeric variables (0, 1, and 2), we found studies' quality mean scores ranging between 0.71 and 2 (with 2 being the highest quality) for each study. According to our interrater judgment of quality, Bachmann et al. [13] and Janssen et al. [50] were the most robust and valid studies with 1.57/2 and 2/2 overall quality scores, respectively.

In sum, the majority of studies (but [12, 13, 50]) were considered high risk on selection biases (random sequence generation, allocation concealment), and all but [50] had a performance bias (blinding of participants and personnel). See Figure 3 and supplementary materials.

## 4. Discussion

### 4.1. Summary of Evidence

In this systematic review, we assessed cognitive and behavioral effects observed in 13 studies using MBIs to alleviate ADHD symptoms and to improve executive function and emotion dysregulation among adults with ADHD. All the studies (100%) showed improvement of ADHD symptoms following an MBI. Researchers have also found a significant improvement on cognitive task performance in post- versus preintervention or with treatment as usual (TAU). For most patients, reduction of ADHD symptoms was maintained at posttreatment (3- to 6-month follow-up). In studies addressing other outcomes, patients reported significant improvements in mindfulness skills, self-compassion, and positive mental health up to the 6-month follow-up.

However, we also found the quality of studies to be variable with a tendency for more recent studies to have less biases. Notably, [50] was given a perfect score according to our application of the Cochrane Collaboration standards. A vast majority of studies were judged as high risk on the performance bias (blinding of participants and personnel), and several had issues with the selection biases (random sequence generation, allocation concealment). As mentioned earlier, it is not habitual, nor always advantageous, to blind personnel or participants in this type of intervention, so the elevated risk for performance and/or detection bias may be inevitable. Attrition bias was found to have high or unclear risk in more than a half of the studies. The reason for dropout of participants was not always clearly specified in those studies, so it is difficult to decide if it might be related to adverse effects or to some discomfort with treatment or instead to some incidental reasons.

Despite the above limitations, most studies (except one) scored well on the detection bias, meaning that the trainers were not involved in the assessment of the participants and therefore could not interfere with the outcomes. Moreover, most studies (except for the two studies being unclear) were found free of suggestion of selective reporting (reporting bias).

In sum, most studies show that mindfulness training or structured programs with mindfulness components appear useful for patients who respond partially or not at all to drug therapy. Indeed, group skills training may be efficacious, acceptable, and feasible for treating ADHD among college students and adult patients. Mindfulness meditation training seems to improve ADHD behavioral symptoms (inattention, hyperactivity, and impulsivity) and some facets of EF and emotion dysregulation. Although these are promising findings to support treatment efficacy of MBIs for ADHD, various biases such as the absence of randomization and lack of a control group may affect the importance of outcomes. Other factors such as those documented in the present study (see Tables 1 and 2) may also impact on the outcomes. For example, the amount of home exercise, type of monitoring of participants' progress, or absence from sessions may also affect the outcomes.

## 5. Conclusions, Future Research, and Limitations

The aim of this systematic review was to look for symptoms and additional indicators of improvement of ADHD mediated through mindfulness interventions. Each study measured many outcomes, namely, executive functions, emotional disturbance, quality of life, and academic performance. Some outcomes were considered to be important (e.g., symptoms), while others were surrogate outcomes (e.g., attention test). Despite its comprehensiveness, this review was not without limitations, mostly because of heterogeneity of available studies. Although all studies included a mindfulness-based intervention for ADHD, there was a substantial variability among them, e.g., difference in sample size and duration of intervention. Future studies and potential meta-analysis should consider these factors.

## Figures and Tables

**Table 1a:** (a) Characteristics of single-group studies: research design and participants

1st author (date)	Research design	Age participant (y/o)	*N*	% males	% ADHD subtypes	% medication status	% comorbid disorders
Hesslinger (2002) [14]	Pre-post	19-44, $\bar{x\;}=31.9$, *s* = 9.0	15	62.2	C = 75, I = 12.5, H = 12.5	37.5 (MPH), 12.5 (other)	37.5 (MDD/Sx), 25 (social phobia), 25 (insomnia)
Morgensterns (2016) [42]	Baseline (T1), posttreatment (T2), 3-month follow-up (T3)	19–63, $\bar{x\;}=37.4$, *s* = 10.4	98	31.6	C = 86.6, I = 13.4	74.7 (atomoxetine+central stimulants), 88.2 (psychoactive drugs)	71.1 (at least one comorbid DSM-IV diagnosis)
Zylowska (2008) [11]	Pre (T1) to post (T2)	$\bar{x\;}=48.5$, *s* = 10.9	24	38	C = 50, I = 42, H = 8	63	83 (mood), 33 (AD), 33 (ODD), 92 (any)

(1) Statistics: n.r. = nonreported; y/o = years old; $\bar{x\;}$ = mean; *s* = standard deviation. (2) ADHD subtypes: ADHD = attention-deficit and hyperactivity disorders; I = inattentive; H = hyperactive; C = combined. (3) Comorbid disorders: AD/Sx = anxiety disorder or symptoms; ODD = oppositional defiant disorder; MDD/Sx = major depressive disorder or symptoms. (4) MPH = methylphenidate.

**Table 1b:** (b) Characteristics of single-group studies: intervention

1st author (date)	Intervention	Intervention length	Therapist	Informants
Hesslinger (2002) [14]	ST-based DBT+mindfulness components	2 h/week, 13 weeks = 26 h	Psychotherapists trained in DBT	Self, objective
Morgensterns (2016) [42]	DBT (elements of acceptance, mindfulness, functional behavioral analysis, psychoeducation)	2 h/week, 14 weeks = 28 h	Two clinical psychologists who are trained in CBT+DBT experienced from previous study phases (T.H.) or had clinical supervision from the experienced group leader	Self
Zylowska (2008) [11]	MAP+psychoeducation	2.5 h/week, 8 weeks = 20 h+daily at-home practice	Experienced mindfulness instructor (D.W.)+ADHD researchers (L.Z. & S.S.)	Self, objective

Intervention: CBT = cognitive behavioral therapy; DBT = dialectical behavior therapy; MAP = mindful awareness program; ST = skills training.

**Table 1c:** (c) Characteristics of single-group studies: measures of outcome

1st author (date)	ADHD symptoms	Cognitive/executive function	Emotional disturbance	Quality of life	Academic performance	Mindfulness
Hesslinger (2002) [14]	ADHD-CL, SCL-16	Fluency, Stroop, DSS, KLT, d2-Test, WMS-R	BDI	n.r.	n.r.	n.r.
Morgensterns (2016) [42]	CADHDSC_SRF: ADHD symptoms (functional impairment, aggression irritability)	n.r.	BDI, BAI, PSS	AAQ-9, AAQoL, KSQ	n.r.	MAAS
Zylowska (2008) [11]	ADHD Rating Scale IV (adults), DSM-IV	ANT, TMT, DST, VOC	BAI, BDI	n.r.	n.r.	n.r.

(1) ADHD symptoms: ADHD-CL = Attention-Deficit Hyperactivity Disorder Checklist; CADHDSC_SRF = Current ADHD Symptom Scale-Self-Report Form; DSM-IV = Diagnostic and Statistical Manual of Mental Disorders, 4th Edition; SCL-16 = Symptom Check List. (2) Cognitive/executive function: ANT = Attention Network Task; d2-Test = selective attention; DSS = Digit Symbol Subtest; DST = Digit Span Test (WAIS-R); KLT = Konzentrations-Leistungs-Test; TMT = Trail Making Test; VOC=vocabulary subtest (WAIS-R); WMS-R = Wechsler Memory Scale-R (mental control, digit span, visual memory span). (3) Emotional disturbance: BAI = Beck Anxiety Inventory; BDI = Beck Depression Inventory; PSS = Perceived Stress Scale. (4) Quality of life: AAQ-9 = Adult Quality of Life Questionnaire; AAQoL = Adult ADHD Quality of Life Questionnaire; KSQ = Karolinska Sleep Questionnaire. (5) Mindfulness: MAAS = Mindful Attention Awareness Scale.

**Table 2a:** (a) Characteristics of between-group studies (age and percentages only for ADHD groups with treatment (Tx)): research design and participants

1st author (date)	Research design	Age (y/o)	*N* of ADHD, HC, Tx, and WL/no/other Tx	% males	% ADHD subtypes	% medication status	% comorbid disorders
Bachmann (2018) [13]	RCT, pre/post	18-65, $\;\bar{x}=40$, *s* = 10.58	40, 0, 21, 19	38	C = 81, I = 19	None (3 months before and during the study)	14 (AD), 57 (Sx), 4 (OC), none (Schizo, BD, SD, AU, SUI/SI, ND, Somato)
Bueno (2015) [44]	N-RCT, pre/post	18-45, $\bar{x}=31.2$, *s* = 7.5	43, 17, 29, 31	54.5	n.r.	69.7 (MPH)	n.r.
Cole (2016) [40]	ADHD-treated vs. ADHD-WL at baseline, post, 3-month and 6-month follow-up, end of treatment	$\bar{x}=36.6$, *s* = 10.02	62, 0, 49, 13	54	C = 73.5, I = 22.5, H = 5	61.22 (MPH), 24.49 (other)	46.94 (MDD, BD, AD, SD, BPD)
Edel (2017) [32]	N-RCT, WL, pre-post	$\bar{x}=33.8$, *s* = 10.1	91, 0, 39, 52	59	C = 69.2, I = 30.8	43.6 (MPH), 38.5 (other)	25.6 (BPD), 30.8 (other PD), 17.9 (social anxiety), 10.3 (MDD/Sx), 5.1 (dysthymia), 2.6 (SD)
Fleming (2015) [35]	RCT, baseline, post, 3-month follow-up	$\bar{x}=21.2$, *s* = 1.67	33, 0, 17, 16	58.8	C = 5.9, I = 88.2 + 5.9 (4 (Sx))	29.4 (MPH), 41.2 (other), 29.4 (none)	AD, MDD/Sx (% n.r.)
Gu (2018) [48]	RCT, ADHD-treated vs. ADHD-WL pre-post, 3-month follow-up	19-24, $\bar{x}=20.2$, *s* = 1.03	54, 0, 28, 26	57.1	C = 6.3, I = 93.3	28.6 (MPH), 42.8 (other), 28.6 (none)	n.r.
Hepark (2019) [45]	RCT, WL	18-65, $\bar{x}=36.5$, *s* = 10	103, 0, 55, 48	38	CAARS-INV: I = 5.2, H = 5.8 *CAARS-SR*: I = 4.3, H = 5.1	46 (MPH), 15 (other), 40 (none)	n.r.
Janssen (2019) [50]	RCT, ADHD-MBCT+TAU vs. ADHD-TAU, baseline, post, 3-month and 6-month follow-up	18+, $\bar{x}=39.7$, s = 11.1	120, 0, 60, 60	47	C = 50, I = 38, H = 8	60	38 (MDD/Sx), 2 (BD), 13 (AD), 70 (Somato), 2 (ED), 2 (dysthymia)
Mitchell (2017) [25]	WL & treatment group, pre-post	18-50, $\bar{x}=40.55$, *s* = 6.83	20, 0, 11, 9	45.5	C = 27.3, I = 72.7	54.5 (“stimulants”)	54.5
Schoenberg (2014) [12]	RCT, WL, pre-post	19-53, $\bar{x}=39.5$, *s* = 9.5	50, 0, 26, 24	37.5	n.r.	38 (MPH), 24 (other), 38 (none)	n.r.

(1) Statistics and design: n.r. = nonreported; y/o = years old; $\bar{x}$ = mean; *s* = standard deviation; HC = healthy control; N-RCT = nonrandomized control trial; RCT = randomized control trial; Tx = treatment; WL = waiting list. (2) ADHD subtypes: ADHD = Attention-Deficit and Hyperactivity Disorders; I = inattentive; H = hyperactive; C = combined. (3) Comorbid disorders: AD/Sx = anxiety disorder or symptoms; AU = autism; BD = bipolar disorder; BPD = borderline personality disorder; ED = eating disorder; MDD/Sx = major depressive disorder or symptoms; ND = neurological disorders; OC = obsessive compulsive disorders. (4) PD = any personality disorder; Somato = somatoform disorder; SD = substance dependence; SUI\SI+suicidality: self-injurious behavior. MPH = methylphenidate.

**Table 2b:** (b) Characteristics of between-group studies: intervention

1st author (date)	Intervention	Intervention length	Therapist	Informants
Bachmann (2018) [13]	MAP or PE	2.5 h/week, 8 weeks = 20 h+daily home practice	n.r.	Self, objective
Bueno (2015) [44]	MAP or no intervention	2.5 h/week, 8 weeks = 20 h+daily home practice	Highly experienced practitioners	Self, objective
Cole (2016) [40]	DBT (+elements of mindfulness) or CBT modules (impulsivity/hyperactivity, attention)	2 h individual psychotherapy+group/week, 12-month period = 96 h+homework assignments	Nurses, psychologists, and psychiatrists, trained in DBT+CBT	Self
Edel (2017) [32]	MBT (+mindfulness component of DBT) or ST (DBT-oriented skills training)	2 h/week, 13 weeks = 26 h	Experienced psychologist working with ADHD+5 y experience in DBT/MBT	Self, expert-rated scale
Fleming (2015) [35]	DBT (+elements of mindfulness) or skills handouts	1.5 h group/week, 8 weeks = 12 h+7 10 min individual coaching/week+90 min group (1st week follow-up)	Group leader (A.P.F.), coleader (L.R.M.), graduate students in clinical psychology with DBT training & intervention, psychologist with experience in ADHD students	Self, objective
Gu (2018) [48]	MBCT	1 h individual/week, 6 weeks = 6 h+30 min self-practice/day workbook psychoeducation	Group leader, psychiatrist specializing in ADHD+8 y experience, MBCT trainers, psychologist with experience in ADHD students	Self, objective
Hepark (2019) [45]	MBDT (+PE)	12-week meditation exercises built up gradually+home practice 30 min/day	Psychiatrist specializing in ADHD (10 y), mindfulness teacher (S.H.) & nurse specialist, Association of Mindfulness-Based Teachers, 150 h education (MBSR)/MBCT	Self, investigator
Janssen (2019) [50]	MBDT (+PE)	2.5 h group/week, 8 weeks = 20 h+6 h silent day+home practice 30 min/day	Mindfulness teachers at different levels of competence	Self, objective
Mitchell (2017) [25]	MAP	2.5 h/week, 8 weeks = 20 h+home practice	Ph.D. clinical psychology	Self, objective
Schoenberg (2014) [12]	MBCT	3 h/week, 12 weeks = 36 h+30-45 min self-practice/day	Psychiatrist specializing in ADHD with 9 y training in MBCT	Self, objective

Intervention: CBT = cognitive behavioral therapy; DBT = dialectical behavior therapy; MAP = mindful awareness program; MBT/MBCT = mindfulness-based training; PE = psychoeducation; ST = skills training.

**Table 2c:** (c) Characteristics of between-group studies: measure of outcome

1st author (date)	ADHD symptoms	Cognitive/executive function	Emotional disturbance	Quality of life	Academic performance	Mindfulness
Bachmann (2018) [13]	CAARS-SR/OR	*n*-back	n.r.	n.r.	n.r.	n.r.
Bueno (2015) [44]	ASRS	ANT, CPT-2	BDI, STAI-T, PANAS-X	AAQoL	n.r.	n.r.
Cole (2016) [40]	ASRS v1.1, BIS-11	n.r.	BDI-II, BHS, STAXI	WHOQoL-BREF, QFS	n.r.	KIMS
Edel (2017) [32]	DSM-IV-(SR/OR)	n.r.	WRI	GSES	n.r.	MAAS
Fleming (2015) [35]	BAARS-IV, BADDS	CPT-2	BAI, BDI-II	AAQoL	GPA	FFMQ
Gu (2017) [48]	CAARS-S:SV	ANT	BAI; BDI-II	VAS	GPA	MAAS
Hepark (2019) [45]	CAARS-INV, CAARS-SR	BRIEF-ASR	BDI-II-NL, STAI	OQ 45.2	n.r.	KIMS
Janssen (2019) [50]	CAARS-INV:SV, CAARS-S: SV	BRIEF-A	n.r.	OQ 45.2, MHC-SF	n.r.	FFMQ-SF, SCF-SF
Mitchell (2017) [25]	Current ADHD Symptom Scale	DEFS, BRIEF-A, ANT, CPT, DST, TMT, WAIS-R	DERS, DTS	n.r.	n.r.	n.r.
Schoenberg (2014) [12]	CAARS-S:SV	CPT-X	n.r.	OQ 45.2	n.r.	KIMS

(2) ADHD symptoms: ASRS (v1.1) = Adult ADHD Self-Report Scale; BAARS-IV = Barkley Adult ADHD Rating Scale-IV; BADDS = Brown ADD Rating Scales; BIS-11 = 11th version of the Barratt Impulsiveness Scale; CAARS-S:SV = Conners' Adult ADHD Self-Rating Scale; CAARS-SV = Conners' Adult ADHD Rating Scales-Screening Version; CAARS-SR = self-report version of the Conners' Adult ADHD Rating Scale; CAARS-INV = investigator rating version of the Conners' Adult ADHD Rating Scale; DSM-IV-(SR/OR) = Diagnostic and Statistical Manual of Mental Disorders, 4th Edition (self-rating/other-rating). (2) Cognitive/executive function: ANT = Attention Network Task; BRIEF-A = Behavior Rating Inventory of Executive Functioning-Adult Version; BRIEF-ASR = Behavior Rating Inventory of Executive Function-Adult Self-Report version; CPT-2 = the Conners' Continuous Performance Test-2nd edition; CPT-X = visual Continuous Performance Task; DEFS = Deficits in Executive Functioning Scale; DST = Digit Span Test (WAIS-R); TMT = Trail Making Test; WAIS-R = Wechsler Adult Intelligence Scale-Revised. (3) Emotional disturbance: BAI = Beck Anxiety Inventory; BDI-II/BDI-II-NL = Beck Depression Inventory (2nd edition); BHS = Beck Hopelessness Scale; DERS = Difficulties in Emotion Regulation Scale; PANAS-X = Affect Schedule-Expanded form; STAI = State-Trait Anxiety Inventory; STAXI = State-Trait Anger Expression Inventory; WRI = Wender–Reimherr Interview. (4) Quality of life: AAQoL = Adult ADHD Quality of Life Questionnaire; GSES = Generalized Self-Efficacy Scale; MHC-SF = Positive Mental Health Short Form; OQ 45.2 = Outcome Questionnaire; QFS = questionnaire of social functioning; VAS = Visual Analog Scale (personal health status); WHOQoL-BREF = World Health Organization Quality of Life. (5) Mindfulness: MAAS = Mindful Attention Awareness Scale; FFMQ = Five Facet Mindfulness Questionnaire; KIMS = Kentucky Inventory of Mindfulness Skills; SCF-SF = Self-Compassion Short Form. (6) Academic performance: GPA = Grade Point Average.

## Appendix

### Full Names of Most Frequently Used Outcome Measures (See Also Tables 1(c)–2(c))

ADHD symptoms measures:

1. ADHD-CL = Attention-Deficit Hyperactivity Disorder Checklist
2. ASRS (v1.1) = Adult ADHD Self-Report Scale
3. BAARS-IV = Barkley Adult ADHD Rating Scale–IV
4. BADDS = Brown ADD Rating Scales
5. BIS-11 = 11th version of the Barratt Impulsiveness Scale
6. CAARS-INV = investigator rating version of the Conners' Adult ADHD Rating Scale; CAARS-S: SV = Conners' Adult ADHD Self-rating Scale; CAARS-SV = Conners' Adult ADHD Rating Scales–Screening Version; CAARS- SR = self-report version of the Conners' Adult ADHD Rating Scale
7. CADHDSC_SRF = Current ADHD Symptom Scale-Self-Report Form
8. DSM-IV (SR/OR) = Diagnostic and Statistical Manual of Mental Disorders, 4th Edition (Self-rating/Other-rating)
9. DSM-IV = Diagnostic and Statistical Manual of Mental Disorders, 4th Edition; SCL-16 = Symptom Check List

Cognitive/EF measures:

1. ANT = Attention Network Task
2. BRIEF-A = Behavior Rating Inventory of Executive Functioning-Adult Version
3. BRIEF-ASR = Behavior Rating Inventory of Executive Function-Adult Self-Report version
4. CPT-2 = The Conners' Continuous Performance Test-2nd edition
5. CPT-X = visual Continuous Performance Task
6. d2-Test = selective attention
7. DEFS = Deficits in Executive Functioning Scale
8. DSS = Digit Symbol Subtest
9. DST = Digit Span Test (WAIS-R)
10. KLT = Konzentrations-Leistungs-Test
11. TMT = Trail Making Test
12. VOC = Vocabulary subtest (WAIS-R)
13. WAIS-R = Wechsler Adult Intelligence Scale-Revised
14. WMS-R = Wechsler Memory Scale-R

Emotional disturbance measures:

1. BAI = Beck Anxiety Inventory
2. BDI = Beck Depression Inventory; BDI-II = Beck Depression Inventory (2nd edition)
3. BHS = Beck Hopelessness Scale
4. DERS = Difficulties in Emotion Regulation Scale
5. PANAS-X = Affect Schedule-Expanded form
6. PSS = Perceived Stress Scale
7. STAI = State-Trait Anxiety Inventory
8. STAXI = State-Trait Anger Expression Inventory
9. WRI = Wender–Reimherr Interview

## Supplementary Materials

Supplementary Materials Full details of analysis of bias for each of individual studies (*n* = 13).
