# Supplementary material for: Behavioral and Cognitive Impacts of Mindfulness-Based Interventions on Adults with Attention-Deficit Hyperactivity Disorder: A Systematic Review
Source: Behav Neurol. 2019 Apr 4;2019:5682050. doi: 10.1155/2019/5682050 (PMC6476147; doi:10.1155/2019/5682050)
Supplement: Supplementary Materials — Full details of analysis of bias for each of individual studies (n = 13). [file 5682050.f1.docx]

**Supplementary Material : Analysis of risk of bias**

| **#1 Study name: Bachmann (2018)** | | |
| --- | --- | --- |
| **Type of bias** | **Judgement** | **Support for judgement** |
| **Random sequence generation (selection bias)** | Low | **Quote**: Eligible patients were randomly assigned to either the PE (n=37)  or MAP (n=37) group. |
| **Allocation concealment (selection bias)** | Low | **Quote:** Randomisation was performed based on a computer-generated allocation sequence (1:1 ratio) by an independent researcher. |
| **Blinding of participants and personnel (performance bias)** | High | **Comment:** no indication that the participants were not aware of the type of Tx.  **Comment: no indication that the trainer was blind to the Tx** |
| **Blinding of outcome assessment (detection bias)** | Low | **Quote:** symptoms were assessed by means of the CAARS self- and blind observer ratings; 27 patients in the PE group and 32 patients in the MAP group were assessed again for ADHD symptom severity via the CAARS self- and blind observer rating.  **Quote**: participants performed a one-back letter task. |
| **Incomplete outcome data (attrition bias)** | Unclear | **Quote**: There were no significant differences between the MAP and PE  group for any of the demographic variables of age, gender and education …no significant differences were found in terms of the ADHD symptoms, subtype, comorbidity or medication.  **Comment:** the final sample consisted of 21/32 ADHD patients who participated in MAP and 19/27 ADHD patients who underwent PE.  **Comment:** 32% of total attrition > 20% |

| **#1 Study name: Bachmann (2018) - cont’d** | | |
| --- | --- | --- |
| **Type of bias** | **Judgement** | **Support for judgement** |
| **Selective outcome reporting (reporting bias)** | Low | **Comment:** All outcome data are reported for scales and subscales. |
| **Other biases**  **(research allegiance, funding, confounds)** | Low | **Quote:** KB, PS, MK, EH, BF, HM, J. and CMT declare that the research was conducted in the absence of any commercial or financial relationships that could be construed as a potential conflict of interest.  **Quote**: The project was partly funded by the Federal Ministry of Education and Research (Bundesministerium für Bildung und Forschung, BMBF, 01GV0606).  **Quote**: Authors’ contributions: KB, PS: Literature search, figures, data analysis, data interpretation, writing; APL, HM, J., CMT: Literature search, data interpretation; MK, EH: Literature search, figures, data collection; BF: Literature search, figures, data collection, data interpretation; SM, AP: Literature search, figures, study design, data collection, data analysis, data interpretation, writing, supervision. |
| **Other limitations** |  | **Quote**: Future research is needed to reveal whether, at a later point in treatment  when mindfulness meditation is applied more automatically, additional significant mindfulness-related brain activation is evoked.  **Quote:** … be of interest to assess the frequency and duration of mindfulness practice to investigate whether those who practice more often exhibit stronger effects and whether a certain intensity of practice is needed …  **Quote**: no healthy control group; … did not implement either a control group with treatment as usual or a nontreatment control group  **Quote**: comorbid disorders may have confounded our results |

| **#2 Study name: Bueno et al. (2015)** | | |
| --- | --- | --- |
| **Type of bias** | **Judgement** | **Support for judgement** |
| **Random sequence generation (selection bias)** | High | **Quote**: We were unable to recruit a sufficient number of participants to allow a randomized study. |
| **Allocation concealment (selection bias)** | High | **Comment:** N-RCT; authors do not provide information about concealment procedures. |
| **Blinding of participants and personnel (performance bias)** | High | **Quote**: Participants could self-select whether they would or not participate in the MAP intervention; It cannot be excluded that the awareness of participants that they would be submitted to the interventions biased the observed effects.  **Quote:** The experimenter was not blind to the Tx.  **Quote**: The material was translated into Portuguese with the permission of the authors, adapted for use in Brazil for both ADHD patients and healthy controls, and was administered by the same highly experienced MAP practitioner.  **Quote**: The authors thank S. L. (not an author) for conducting the mindfulness practices. |
| **Blinding of outcome assessment (detection bias)** | Low | **Comment**: Use of self-report: Subjective Questionnaires and objective measures. |
| **Incomplete outcome data (attrition bias)** | Low | **Quote**: Two ADHD participants and two HC allocated to the MAP intervention dropped out of the study for personal reasons. Their data were excluded from the analyses. Three control participants two from the MAP group) did not attend the reevaluation after the intervention period, and their data were excluded. Our final sample consisted of twenty-one ADHD patients (11 men) and eight controls (3 men) who participated in MAP and twenty-two ADHD patients (12 men) and nine controls (4 men) who underwent no intervention.  **Comment:** Diagnosis groups are not equivalent; Tx. and no intervention are almost equivalent.  5 dropouts on 48 ADHD: 3 MAP and 2 no intervention;  3 dropouts on 20 HC: 2 MAP and 1 no intervention.  11.7% of total attrition < 20%. |
| **#2 Study name: Bueno et al. (2015) - cont’d** | | |
| **Type of bias** | **Judgement** | **Support for judgement** |
| **Selective outcome reporting (reporting bias)** | Low | **Comment:** All outcome data are reported for scales and subscales. |
| **Other biases**  **(research allegiance, funding, confounds)** | Low | **Quote:** The authors declare no conflict of interests with respect to the authorship and/or publication of this paper.  This research was supported by […] nonprofit organizations that sponsor research in Brazil. The authors thank Stephen Little (S.L.) for conducting the mindfulness practices.  **Comment:** S.L. not an author. |
| **Other limitations** |  | **Quote**: […] The small number of control participants.  **Quote**: The experimenter was not blind to the treatment. However, we believe that this did not compromise our data because the ADHD patients and controls who agreed to participate in the intervention and those who did not did not differ in terms of demographic variables or IQ or on any subjective measure except inattention.  **Quote**: It can also not be excluded that the awareness of participants that they would be submitted to the intervention biased the observed effects. However, we would expect this to influence only subjective measures and not the attentional ones, which were also improved, suggesting that our data do not reflect pure expectation effects.  **Quote**: The possibility that patients with different ADHD subtypes would have reacted differently to the MAP intervention cannot be excluded  **Quote**: Age and gender specific effects must also be investigated, as should the impact of MAP on non-medicated and medicated patients. |

| **#3 Study name: Cole et al. (2016)** | | |
| --- | --- | --- |
| **Type of bias** | **Judgement** | **Support for judgement** |
| **Random sequence generation**  **(selection bias)** | High | **Quote:** This study was a non-randomized study (NRCT) on a small sample of patients. The design of the study probably leads to a recruitment bias among patients enrolled in the program or placed on the WL. |
| **Allocation concealment (selection bias)** | High | **Quote**: For the purpose of this study, we recruited patients suffering from ADHD who are being treated in our specialized center for the treatment of adult ADHD.  **Comment:** N-RCT and authors do not provide information about concealment procedures. |
| **Blinding of participants and personnel (performance bias)** | High | **Quote:** The therapists are nurses, psychologists or psychiatrists, all trained in DBT and CBT.  **Comment:** No blinding of participants or facilitators. |
| **Blinding of outcome assessment (detection bias)** | Low | **Comment**: self-rated questionnaires. |
| **Incomplete outcome data (attrition bias)** | Low | **Quote:** 7 of the ADHD patients following the psychotherapeutic treatment dropped out during the one-year programme (14.29%). None of the clinical and demographic baseline characteristics were associated with dropping-out.  % attrition < 20% according to criteria of Campbell. |
| **Selective outcome reporting**  **(reporting bias)** | Unclear | **Comment**: Only significant outcomes are reported:  QFS & WHOQoL-BREF not reported. |
| **#3 Study name: Cole et al. (2016) - cont’d** | | |
| **Type of bias** | **Judgement** | **Support for judgement** |
| **Other biases**  **(research allegiance, funding, confounds)** | Low | **Quote:** Conflict of interest: None to declare.  **Comment:** No indication of researchers’ role in the development, adaptation and/or delivery of the interventions. |
| **Other limitations** |  | **Quote:** small sample size, especially for the waiting-list controls; it might help explain the absence of significant differences between the groups. We are therefore unable to exclude the idea that observed improvements in ADHD patients undergoing the psychotherapeutic intervention can be better explained by a phenomenon such as regression to the mean. Nevertheless, our results are in line with previous studies in the field. Furthermore, the fact that controls worsen on most of the assessed dimensions during the year spent on the waiting list supports the idea that our approach is a useful intervention for adult ADHD.  **Quote:** patients were only monitored over a one-year period, and we cannot state whether our intervention is associated with a long-term improvement of ADHD symptoms. |

| **#4 Study name: Edel et al. (2017)** | | | |
| --- | --- | --- | --- |
| **Type of bias** | **Judgement** | | **Support for judgement** |
| **Random sequence generation**  **(selection bias)** | | High | **Quote**: Adults with ADHD were non-randomly (N-RCT) assigned to and treated within or STG. |
| **Allocation concealment**  **(selection bias)** | | High | **Comment:** N-RCT and authors do not provide information about concealment procedures. |
| **Blinding of participants and personnel (performance bias)** | | High | **Quote**: Adults with ADHD […] recruited from our outpatient unit for adults with ADHD.  **Quote:** The mindfulness-based training was created by the authors and based on Jon Kabat-Zinn’s Mindfulness-Based Stress Reduction (MBSR) program.  **Quote:** Raters and evaluators were not blind to the assignment of participants to the two interventions.  **Comment:** Blinding to researchers uncertain.  **Quote:** Group allocation followed individual requirements, such as patient- or therapist-estimated, need to improve organizational skills, patients’ preferences, or individual scheduling issues.  **Quote:** Sessions were conducted by an experienced psychologist and psychotherapist (T.H.) and an experienced consultant in psychiatry and psychotherapy (M.A.E.).  **Comment:** T.H. & M.A.E are also authors/researchers.  **Comment:** No blinding of participants or personnel. |
| **Blinding of** **outcome assessment**  **(detection bias)** | | High | **Quote**: Raters and evaluators were not blind to the assignment of participants to the two interventions.  **Quote**: self-designed Likert type scales + other-ratings (partners, relatives).  **Comment**: mix of self-rated and other-rating.  **Quote**: expert-rated scale … with an interview part. |
| **Incomplete outcome data (attrition bias)** | | Unclear | **Comment**: not addressed by the authors. |

| **#4 Study name: Edel et al. (2017) - cont’d** | | |
| --- | --- | --- |
| **Type of bias** | **Judgement** | **Support for judgement** |
| **Selective outcome reporting**  **(reporting bias)** | Low | **Comment:** All outcome data are reported for scales and subscales. |
| **Other biases**  **(research allegiance, funding, confounds)** | Low | **Quote:** The author(s) declared no potential conflicts of interest with respect to the research authorship, and/or publication of this article.  The author(s) received no financial support for the research, authorship, and/or publication of this article. |
| **Other limitations** |  | **Quote:** […] lack of matching groups concerning BPD (and other comorbidities). Regarding group assignment, therapists possibly felt that that MBTG might be more suitable for the patients with obvious emotional instability, and the SGT would better address organizational problems. […] Perhaps some patients with BPD comorbidities preferred the novel mindfulness based approach, which might be another reason for this bias. Moreover, we did not control for depression or anxiety.  **Quote:** the new mindfulness based intervention lacked impact and structured treatment monitoring. Regarding impact, the use of a CD audio program with (instructions for) formal meditation exercises […] would have been helpful. As to structured monitoring, the administration of electronic diaries using handheld computers […] may enhance both the accuracy of the follow up assessment and treatment consistency […] a flowchart to assess participant progress and an explicit assessment of the reasons for missing sessions […] could improve patients’ commitment, adherence, and outcomes.  **Quote:** MAAS […] have been criticized for measuring a component of one’s daily attention rather than the essence of mindfulness. |

| **#5 Study name: Fleming et al. (2015)** | | |
| --- | --- | --- |
| **Type of bias** | **Judgement** | **Support for judgement** |
| **Random sequence generation (selection bias)** | Unclear | **Quote**: Participants were stratified by a median split on ADHD inattentive symptoms and randomly assigned to receive either DBT group skills training or self-guided skills training handouts.  **Quote**: Participants were randomly assigned to treatment condition.  **Comment:** No sufficient description of the method used to generate the allocation sequence. |
| **Allocation concealment (selection bias)** | Unclear | **Comment:** random assignment but authors did not provide information about concealment procedures. However, both groups include an active intervention, which might conceal the allocation. |
| **Blinding of participants and**  **personnel (performance bias)** | High | **Quote**: Participants were randomly assigned to treatment condition and were assessed at pre-treatment, post-treatment, and 3-month follow-up by an interviewer who was blind to participant condition.  **Comment:** blinding of assessor but no blinding of participants.  **Quote:** The intervention was delivered by a group leader (A.P.F.) and co-leader (L.R.M.) who were both advanced graduate students in child clinical psychology.  **Comment:** A.P.F. and L.R.M. are both authors of the paper and are were blinded to the participant condition. |
| **Blinding of outcome assessment (detection bias)** | Low | **Comment**: Use of self-report: Subjective Rating Questionnaires and objective measure |

| **#5 Study name: Fleming et al. (2015) - cont’d** | | |
| --- | --- | --- |
| **Type of bias** | **Judgement** | **Support for judgement** |
| **Incomplete outcome data (attrition bias)** | Low | **Quote**: The intent-to-treat sample included 17 (10 men) and 16 participants (9 men) DBT group skills training and self-guided SH, respectively. One participant dropped out of DBT after four sessions and did not complete the post-treatment or follow-up assessments; all other participants completed treatment and the three study assessments. Missing data from this participant were imputed conservatively using the last observation carried forward (LOCF) method.  **Quote:** Participants […] were required to meet *Diagnostic and Statistical Manual of Mental Disorders* […] criteria for ADHD in adulthood, including symptom onset by age 12 and functional impairment in multiple domains.  **Comment:** The two groups are almost equivalent in terms of gender, diagnosis and ADHD symptoms.  2 dropout on 19 in DBT group and 1 was not retained for assessment;  0 dropout on 16 in skills training group;  5.7% of total attrition < 20%. |
| **Selective outcome reporting (reporting bias)** | Low | **Comment:** Data for primary and secondary outcomes were reported for all used scales, but some subscales were not reported (e.g., FFMQ). However, no indication that omitting to report subscales induced a bias. |
| **Other biases (research allegiance, funding,**  **confounds)** | Unclear | **Quote**: The author(s) declared no potential conflicts of interest with respect to the research, authorship, and/or publication of this article.  **Quote**: The author(s) disclosed receipt of the following financial support for the research, authorship, and/or publication of this article: This study was supported by the University of Washington-Robert C. Bolles Doctoral Research Fellowship.  **Comment:** Researchers delivered the intervention. |

| **# 6 Study name: Gu et al. (2018)** | | | |
| --- | --- | --- | --- |
| **Type of bias** | **Judgement** | | **Support for judgement** |
| **Other limitations** |  | **Quote**: The small sample size of this pilot randomized trial does not provide the statistical power needed to detect small-to-moderate effect sizes or to assess mediators and moderators of treatment response.  **Quote**: This study cannot rule out therapist effects or non-specific factors of group psychotherapy. | |
| **Random sequence generation**  **(selection bias)** | Unclear | | **Quote**: Participants were randomly allocated to either. MBCT condition or WL control group. Groups were balanced taking account of gender, age, ADHD subtypes, and medication status.  **Comment:** No sufficient description of the method used to generate the allocation sequence. |
| **Allocation concealment**  **(selection bias)** | High | | **Comment:** random assignment but authors did not provide information about concealment procedures. In addition, one of the groups is WL, making it impossible to conceal the allocation. |
| **Blinding of participants and personnel (performance bias)** | High | | **Quote:** The study population consisted of 27 undergraduate students. […] seeking treatment.  **Quote**: [control group] would be offered MBCT at the end of the study (i.e., patient preference).  **Comment:** No blinding of participants.  **Quote**: The intervention was delivered by a group leader and co-leader who were psychiatrists specializing in ADHD with 8 years’ experience as MBCT trainers. Intervention was supervised by a licensed psychologist with experience in assessment and treatment of college students with ADHD.  **Comment:** blinding to researchers uncertain. |
| **Blinding of outcome assessment**  **(detection bias)** | Low | | **Quote**: Participants randomly assigned to MBCT group and WL group were assessed […] by an interviewer who was blind to participant condition.  **Comment**: Use of self-report: Subjective Rating Questionnaires and objective measures. |
| **# 6 Study name: Gu et al. (2018) - cont’d** | | | |
| **Type of bias** | **Judgement** | | **Support for judgement** |
| **Incomplete outcome data**  **(attrition bias)** | Low | | **Quote:** Two participants dropped out of MBCT after six sessions and did not complete the post-treatment or follow-up assessments; all other participants completed treatment and the three study assessments.  **Comment:** Small and non-significant attrition between groups. |
| **Selective outcome reporting**  **(reporting bias)** | Low | | **Comment**: All outcome data are reported for scales and subscales. |
| **Other biases**  **(research allegiance, funding, confounds)** | Low | | **Quote:** The author(s) declared no potential conflicts of interest with respect to the research, authorship, and/or publications of this article.  **Quote:** The author(s) received no financial support for the research, authorship, and/or publication of this article. |
| **Other limitations** |  | | **Quote:** The majority of the sample was Chinese students who were recruited through general psychology courses […] these students may not be representative of the college student population.  **Quote:** The sample size was too small for valid analyses.  **Quote:** Potential outcome variables associated with ADHD in college students were not systematically assessed, such as poorer quality of life, self-injurious behaviors, increased cigarette smoking, alcohol and drug dependency, and severity of work performance difficulties.  **Quote:** More heterogeneous samples is needed, both demographically and clinically.  **Quote:** Co-existent anxiety and depression disorders were not systematically assessed, […] the presence of which might affect the efficacy of the treatment.  **Quote:** It is probable that participants may not have reported information honestly. More precise reporting procedures might be used in future research.  **Quote:** Future research should address the impact of potential factors on treatment outcome, such as patient self-efficacy and therapeutic alliance. |

| **#7 Study name: Hepark et al. (2019)** | | |
| --- | --- | --- |
| **Type of bias** | **Judgement** | **Support for judgement** |
| **Random sequence generation**  **(selection bias)** | Unclear | **Quote**: Participants were randomly allocated to mindfulness training (MBCT) or waiting list (WL) control condition by an independent researcher.  **Comment:** No sufficient description of the method used to generate the allocation sequence. |
| **Allocation concealment**  **(selection bias)** | High | **Comment:** Random assignment but authors did not provide information about concealment procedures. In addition, one of the groups is WL, making it impossible to conceal the allocation. |
| **Blinding of participants and personnel (performance bias)** | High | **Comment:** The participants do not appear to be blinded. |
| **Blinding of outcome assessment**  **(detection bias)** | Low | **Quote:** The clinical interviews were conducted single blindly by a psychiatrist.  **Comment**: The clinical assessor was blinded  **Comment**: Use of self-report: Subjective Rating Questionnaires and objective measures. |
| **Incomplete outcome data**  **(attrition bias)** | Unclear | **Quote:** Of the 53 patients randomized to the MBCT group, 12 (23%) did not complete the study, whereas 5 (11%) of 47 patients randomized to the WL group did not complete the post-assessment.  **Quote:** No significant differences were found between completers and non-completers of the mindfulness training on age, sex, use of medication, and clinical characteristics at baseline, except for a trend for patients with more depressive symptoms to drop out more often.  **Quote**: Treatment effects could have been overestimated as they only applied to patients who attended at least six MBCT sessions. To provide a more conservative estimate of the treatment effect, we performed ITT analyses with imputation of missing data according to LOCF. In addition, the smaller sample size at the end of the study might have led to Type II errors. |
|  |  |  |

| **#7 Study name: Hepark et al. (2019) - cont’d** | | |
| --- | --- | --- |
| **Type of bias** | **Judgement** | **Support for judgement** |
| **Selective outcome reporting**  **(reporting bias)** | Low | **Comment:** All outcome data are reported (including all scales and subscales). |
| **Other biases**  **(research allegiance, funding, confounds)** | Low | **Quote:** The author(s) declared the following potential conflicts of interest with respect to the research, authorship, and/or publication of this article: Cornelis C. Kan has also been a member of the advisory board and consultancy team of Eli Lilly BV and was a speaker at the Adult ADHD Academy of Eli Lilly. |
| **Other limitations** |  | **Quote**: The current research did not take into account comorbidity with other psychiatric disorders, despite the considerable amount of literature documenting the comorbidity between ADHD and, for example, mood disorders, substance abuse, and personality disorders. |

| **#8 study name: Hesslinger et al. (2002)** | | |
| --- | --- | --- |
| **Type of bias** | **Judgement** | **Support for judgement** |
| **Random sequence generation**  **(selection bias)** | High | **Quote**: ...eleven patients consecutively seen at our outpatient clinic who fulfilled the above-mentioned criteria. Of these eleven patients, eight agreed to participate .  **Comment:** N-RCT. |
| **Allocation concealment**  **(selection bias)** | High | **Comment:** authors do not provide explicit information about concealment procedures, but the control group (initial 7 and 3 completers) were on the waiting list. |
| **Blinding of participants and personnel (performance bias)** | High | **Comment:** The participants do not appear to be blinded and there is no information provided about blinding the personnel. |
| **Blinding of outcome assessment**  **(detection bias)** | Low | **Comment**: Use of self-report: Subjective Rating Questionnaires and objective measures. |
| **Incomplete outcome data**  **(attrition bias)** | Unclear | **Quote:** This is an exploratory pilot study and, therefore, like in psychopharmacological pilot studies, the number of cases is very small. The control group is clearly compromised by the high dropout rate. Thus, we do not want to comment on the differences in outcome between the treatment and control group.  **Quote:** Given that lack of persistence is one of the symptoms of ADHD, it is remarkable that there were no dropouts and no problems with unpunctuality or missing of sessions in our treatment group.  **Comment:** There was a significant attrition rate for the control group (4 out of 7), but the control group was not used to draw final conclusions (please see quotes below) There was no attrition rate for the pre-post comparisons in the experimental group (8 ADHD patients). |

| **#8 study name: Hesslinger et al. (2002) - cont’d** | | |
| --- | --- | --- |
| **Type of bias** | **Judgement** | **Support for judgement** |
| **Selective outcome reporting**  **(reporting bias)** | Low | **Comment:** All outcome data are reported. |
| **Other biases**  **(research allegiance, funding, confounds)** | Unclear | **Comment**: No funding sources reported, nothing about conflict of interest reported |
| **Other limitations** |  | **Comment:** Small and heterogeneous sample.  **Quote**: Comorbid disorders were recurrent depressive disorder (3 patients), social phobia (2 patients) and insomnia (2 patients). |

| **#9 study name: Jansenn et al. (2018)** | | |
| --- | --- | --- |
| **Type of bias** | **Judgement** | **Support for judgement** |
| **Random sequence generation**  **(selection bias)** | Low | **Quote**: Participants were randomly assigned to MBCT + TAU (n = 60) … or TAU only (n = 60)  **Quote**: Randomisation was stratified by centre, after which block randomisation with varying predefined block sizes was used combined with minimisation for use of medication for ADHD (yes/ no); previous participation in a psychoeducation training (yes/ no); gender and ADHD subtype (combined/inattentive/hyperactive– impulsive/not otherwise specified). |
| **Allocation concealment**  **(selection bias)** | Low | **Quote:** Random assignment to MBCT or TAU was performed by a website  specifically developed for this study by an independent statistician.  The researcher was blind for the block sizes and filled-out the online form. |
| **Blinding of participants and personnel (performance bias)** | Low | **Quote**: Randomisation took place after enrolment, but participants were not informed about the assigned condition until after completion of T0 [baseline].  **Quote:** To ensure the blinding of the interviewers, participants were instructed not to share information about allocation with the interviewer. |
| **Blinding of outcome assessment**  **(detection bias)** | Low | **Quote**: Blinded assessments by a psychiatrist or specialist nurse took place at baseline (T0), post-treatment (T1), 3 (T2) and 6 (T3) months follow-up.  **Quote**: The investigator-rated screening version of CAARS-INV: SV was used by blinded clinicians to assess ADHD symptoms at each time point.  **Quote**: The following self-report questionnaires were administered online as secondary outcomes at each time point. |
|  |  |  |

| **#9 study name: Janssen et al. (2018 - cont’d** | | |
| --- | --- | --- |
| **Type of bias** | **Judgement** | **Support for judgement** |
| **Incomplete outcome data**  **(attrition bias)** | Low | **Quote:** At baseline, there were no significant differences in demographic and clinical characteristics between both groups.  **Quote**: From T0 to T1, […] more participants in the MBCT + TAU group than in the TAU group kept their medication stable.  **Quote:** Within the MBCT + TAU group, participants who dropped out of MBCT (n = 9; 15%) were less likely to use ADHD medication at T0 than MBCT completers. There were no differences in characteristics between those with missing data at T1 on all outcomes (n = 7) and those included in at least one of the Intention To Treat analyses at T1 (n = 113).  **Quote**: Clinician-rated ADHD symptoms at T1 were not predicted by gender; ADHD subtype; use of ADHD medication; comorbid depressive disorder, and comorbid anxiety disorder. Similar results were found for clinician-rated ADHD symptoms over the course of the 6-month follow-up period and in the Per Protocol sample. |
| **Other biases**  **(research allegiance, funding, confounds)** | Low | **Quote**: The research team declares it had no part in developing the original MBCT programme. The team does not gain income from the sale of books on MBCT, nor does it gain income from giving lectures or workshops about it. AS is the founder and clinical director of the Radboudumc Centre for Mindfulness. LJ and MS are affiliated with this centre. JB has been in the past 4 years a consultant to […] Eli Lilly,Lundbeck, Shire, Medice and Servier. He is not an employee of any of these companies and not a stock shareholder of any of these companies. He has no other financial or material support […]. CK has been a member of the advisory board and consultancy team of Eli Lilly BV. The other authors declare that they had no competing interests. |
| **Other limitations** |  | **Comment:** Small and heterogeneous sample.  **Quote**: Comorbid disorders were recurrent depressive disorder (3 patients), social phobia (2 patients) and insomnia (2 patients). |

| **#10 Study name: Mitchell et al. (2017)** | | |
| --- | --- | --- |
| **Type of bias** | **Judgement** | **Support for judgement** |
| **Random sequence generation**  **(selection bias)** | Unclear | **Quote**: Participants were stratified by ADHD medication status and randomized to a treatment or waitlist group.  **Comment:** No sufficient description of the method used to generate the allocation sequence. |
| **Allocation concealment**  **(selection bias)** | High | **Comment:** random assignment but authors did not provide information about concealment procedures. In addition, one of the groups is WL, making it impossible to conceal the allocation. |
| **Blinding of participants and personnel (performance bias)** | High | **Quote**: Practices (MAPs) for ADHD program was administered by a PhD-level clinical psychologist.  **Comment:** no description of blinding of assessors or participants. |
| **Blinding of outcome assessment**  **(detection bias)** | Unclear | **Quote:** Assessments of response to treatment included (a) self-report rating scales and clinician-administered interviews completed in the laboratory, (b) EF laboratory tasks, and (c) self-report rating scales completed *via* electronic diary outside of the laboratory.  **Quote:** ADHD symptoms were assessed in the laboratory with the self-report and unblinded clinician rating versions of the Current ADHD Symptoms Scale.  **Quote:** Raters were aware of group status.  **Comment**: Assessors were not blind during clinical rating interviews. |

| **#10 Study name: Mitchell et al. (2017)** | | |
| --- | --- | --- |
| **Type of bias** | **Judgement** | **Support for judgement** |
| **Incomplete outcome data**  **(attrition bias)** | Low | **Quote**: All participants randomized to the treatment group completed the study, whereas 2 of 11 in the waitlist group did not complete the study (see above).  **Comment:** The two groups are almost equivalent in terms of diagnosis, similar in terms of ADHD medication, but somewhat different in terms of gender composition (5 males and 6 females in the treatment group versus 3 males and 6 females in the waitlist control group).  0 dropout on 11 in mindfulness group;  2 dropout on 11 in the waitlist group;  9.1% of total attrition < 20%. |
| **Selective outcome reporting**  **(reporting bias)** | Low | **Comment:** Data for primary and secondary outcomes were reported for all used scales and subscales. |
| **Other biases**  **(research allegiance, funding, confounds)** | Unclear | **Quote:** The author(s) declared the following potential conflicts of interest with respect to the research, authorship, and/or publication of this article: In the past 2 years, Dr. Kollins has received support and/or consulting fees from the following: Addrenex/Shionogi, Akili Interactive, NIH/NIDA, Otsuka, Pfizer, Purdue Canada, Rhodes, Shire, Sunovion, and Supernus.  **Quote**: The author(s) disclosed receipt of the following financial support for the research, authorship, and/or publication of this article: This study was primarily supported by the American Professional Society of ADHD and Related Disorders/Pond Family Foundation (J.T.M.). Additional funding support was provided by the National Institute of Drug Abuse. |

| **#10 Study name: Mitchell et al. (2017) - cont’d** | | |
| --- | --- | --- |
| **Type of bias** | **Judgement** | **Support for judgement** |
| **Other limitations** |  | **Quote**: The final sample size was small. […] future studies need to include larger sample sizes to ensure greater statistical power.  **Quote**: Mediators and moderators were not considered. For instance, some have argued that ADHD subtypes represent qualitatively different disorders, which would suggest that findings from the current study may vary as a function of ADHD subtype status. Additional factors, such as gender, comorbidity, or race/ethnicity…  **Quote**: The comparison group was a waitlist condition. An active treatment comparison group should be conducted in future studies […] an active treatment comparison group would address whether any improvements were derived from participating in a therapeutic group.  **Quote**: The long term effects of mindfulness meditation training were not considered  **Quote**: in terms of null results involving the EF laboratory tasks, a limited number of tasks were administered.  **Quote**: Corrections for multiple comparisons were not made given the preliminary nature of this study.  **Quote**: Relatedly, a medication washout period should be considered.  **Quote**: The current study attempted to assess outcome variables from  multiple sources (i.e., self-report and clinician) and methods  of assessment (i.e., rating scale, interview, laboratory task performance, EMA). However, not all outcome variables were assessed accordingly. For example, emotion dysregulation was assessed only via two self-report rating scales.  **Comment:** Mindfulness was not measured; no description of the mindfulness training of the facilitator of the mindfulness group. |

| **#11 Study name: Morgenstern et al*.* (2016)** | | |
| --- | --- | --- |
| **Type of bias** | **Judgement** | **Support for judgement** |
| **Random sequence generation**  **(selection bias)** | High | **Quote**: Ninety eight adults (out of 102) with ADHD were allocated to the  treatment.  **Comment:** NRCT study. |
| **Allocation concealment**  **(selection bias)** | High | **Quote:** The participants were recruited from […] clinics by the clinical staff. They were invited to an individual interview with one of the group leaders.  **Comment:** NRCT study. |
| **Blinding of participants and personnel**  **(performance bias)** | High | **Comment:** Neither the participants nor personnel appear to be blinded. |
| **Blinding of outcome assessment**  **(detection bias)** | Low | **Comment**: Use of self-report: Subjective Rating Questionnaires and objective measures. |
| **Incomplete outcome data**  **(attrition bias)** | High | **Quote:** Adults with ADHD allocated to intervention (n=98); Underwent intervention (n=94); Did not undergo allocated intervention (did not come  to first session, n=4); Post-treatment assessment (T2) n= 72; Assessment at three-month follow-up, T3 (n= 58) (Completed T3 but missing T2 (n=2))  **Quote:** Treatment completion was acceptable (80 % attended at least two-thirds of the sessions; the mean attendance among completers was 11.48 session out of 14). However, on applying a more rigorous rule for treatment completion (attendance at 75 % of the sessions), the completion rate dropped to 60%.  **Comment**: 23.4% at T2 and 38% at T3 > 20%. |
| **Selective outcome reporting**  **(reporting bias)** | Low | **Comment:** All outcome data were reported (but the number of participants varied between the three time points, i.e., baseline, post-treatment and follow-up). |
| **#11 Study name: Morgenstern et al*.* (2016) - cont’d** | | |
| **Type of bias** | **Judgement** | **Support for judgement** |
| **Other biases**  **(research allegiance, funding, confounds)** | Unclear | **Comment:** Psychologists who were group leaders are not among the researchers.  **Quote:** Financial support was provided through the Regional Agreement on Medical Training and Clinical Research (ALF) between the Stockholm County Council and Karolinska Institute, as well as the foundation Psykiatrifonden.  **Quote:** Two of the authors are also authors of the Swedish version of the treatment manual. |
| **Other limitations** |  | **Quote:** Compared to many of the previous studies, the present study group had a lower educational level, as well as employment status, a higher level of comorbidity and included persons with lower IQ levels, i.e., individuals with marginal mental retardation who are usually excluded from psychotherapy trials.  **Quote**: changes in concurrent treatments (e.g., medication status) were not systematically assessed.  **Quote:** Outcome measures were based solely on self-report scales and did not include clinician observations. Self-report measurements may be associated with exaggeration or under-reporting of symptoms due to difficulties in analyzing own behavior. However, some of the scales used in this study have previously shown high correlations between investigator-reported and self-reported ADHD symptoms. |

| **# 12 Study name: Schoenberg et al. (2014)** | | |
| --- | --- | --- |
| **Type of bias** | **Judgement** | **Support for judgement** |
| **Random sequence generation**  **(selection bias)** | Low | **Quote**: Sixty-one adult ADHD patients… 32 randomly allocated to the treatment condition (MBCT), and 29 to a wait-list (WL) control group.  **Quote:** Randomization (random number tables) was conducted prior to pre/T1 data collection. |
| **Allocation concealment**  **(selection bias)** | Low | **Quote:** Randomization (random number tables) was conducted prior to pre/T1 data collection. |
| **Blinding of participants and personnel**  **(performance bias)** | High | **Comment:** The participants do not appear to be blinded and there is no information provided about blinding of the trainers. |
| **Blinding of outcome assessment**  **(detection bias)** | Low | **Comment**: Use of self-report: Subjective Rating Questionnaires and objective measures |
| **Incomplete outcome data**  **(attrition bias)** | High | **Quote:** 11 patients (6 MBCT; 5 WL) did not attend the T2/post testing session… Leaving 50 participating patients for the present study; 26 randomly allocated to the MBCT, and 24 to the WL.  **Quote:** The statistically viable *N* = 44 sample (24 MBCT vs. 20 WL) was  matched between groups for Age … Sex and Medication Status.  **Quote:** Of the remaining N = 44, complete clinical datasets were not available for two patients (1 MBCT, 1 WL); in one case the pre/T1, the other the post/T2, questionnaires were not completed at the time of testing due to practical/time constraints.  **Comment:** There is a reduction from the initial 61 recruited participants to 44 participants included in the final analysis: 28% >20%. |
| **Selective outcome reporting**  **(reporting bias**) | Unclear | **Quote:** Due to the various findings analysed vs. reporting length constraints, non-significant results are not explicitly reported.  **Comment:** Several behavioral data, apparently all clinical effects and all mindfulness skills are reported. |
| **# 12 Study name: Schoenberg et al. (2014) - cont’d** | | |
| **Type of bias** | **Judgement** | **Support for judgement** |
| **Other biases**  **(research allegiance, funding, confounds)** | Low | **Quote:** This research was supported by BrainGain SmartMix Programme of the Netherlands Ministry of Economic Affairs and Netherlands Ministry of Education, Culture and Science.  **Comment**: No conflict of interest reported. |
| **Other limitations** |  | **Quote**: The lack of an active control group is a significant methodological constraint. The inclusion of an additional medication control group in the present study would be methodologically advantageous to examine the inferred hypothesis that MBCT has similar effects on pertinent neurotransmission systems in ADHD as pharmacology.  **Quote:** Over half the patients were on psychotropic medication. Albeit, medicated patients were equally dispersed within each group.  **Quote:** Self-report CAARS is not ideal to gauge ADHD symptoms. Ergo, lack of an objective assessment is limiting, although, the self-reported surveys were aimed to supplement the primary ERP and behavioural measures. |

| **# 13 Study name: Zylowska et al. (2008)** | | |
| --- | --- | --- |
| **Type of bias** | **Judgement** | **Support for judgement** |
| **Random sequence generation**  **(selection bias)** | High | **Comment**: NRCT |
| **Allocation concealment**  **(selection bias)** | High | **Comment:** NRCT |
| **Blinding of participants and personnel**  **(performance bias)** | High | **Comment:** NRCT |
| **Blinding of outcome assessment**  **(detection bias)** | Low | **Comment**: Use of self-report: Subjective Rating Questionnaires and objective measures. |
| **Incomplete outcome data**  **(attrition bias)** | High | **Quote:** 75% of adults [87 % adolescents] completed the study.  **Quote:** Four adults dropped out after one session (unknown reason for dropout) one dropped out after four sessions (family emergency), and one failed to complete the post intervention assessment (illness). There were no differences between completers and dropouts based on ADHD sub-type, self-reported severity of AD.  **Comment**: 25% attrition > 20%.  **Comment**: outcome data for adults + adolescents together. |
| **Selective outcome reporting**  **(reporting bias** | Low | **Comment:** All outcome data reported (outcome data for adults + adolescents together). |

| **# 13 Study name: Zylowska et al. (2008) - cont’d** | | |
| --- | --- | --- |
| **Type of bias** | **Judgement** | **Support for judgement** |
| **Other biases**  **(research allegiance, funding, confounds)** | Unclear | **Quote**: The mindfulness training was adapted to meet the unique challenges of ADHD symptoms.  **Comment:** Presumably done by the researchers.  **Quote:** This study was supported by the Robert-Wood Johnson Foundation Clinical Scholars Grant and Norman Cousin’s Center for Psychoneuroimmunology Fellowship Grant to Dr. Zylowska and in part by National Institute of Mental Health MH058277 to Dr. Smalley. |
| **Other limitations** |  | **Quote:** During the course of the study, one adult reported adding modafinil to his medication regimen, one adult reported reducing his or her methylphenidate dose, and one adult reported starting atomoxetine.  **Quote**: Because of the naturalistic type of this pilot study, everyone who completed at least 4 weeks of training and completed the post intervention assessment was included in the analyses. On average, participants attended seven of the eight sessions, with a range of six to eight.  **Comment:** variable length of attendance to Tx.  **Quote:** small sample size; majority of our participants were female, […] white, educated, and from medium to high socioeconomic status. […] average to high average IQ range. […] our sample included several adults with “probable ADHD” and had more lifetime mood disorders and less lifetime oppositional defiant disorder than most ADHD adult.  **Quote:** reliance on self-report measures of psychiatric symptoms […] multi-informant measures would be important to confirm the current preliminary results. |
